# Breath of Life: The Respiratory Vagal Stimulation Model of Contemplative Activity

**DOI:** 10.3389/fnhum.2018.00397

**Published:** 2018-10-09

**Authors:** Roderik J. S. Gerritsen, Guido P. H. Band

**Affiliations:** ^1^Institute of Psychology, Cognitive Psychology, Faculty of Social and Behavioural Sciences, Leiden University, Leiden, Netherlands; ^2^Leiden Institute for Brain and Cognition, Leiden University, Leiden, Netherlands

**Keywords:** meditation, mind-body exercises, mindfulness, respiration, vagus nerve, heart rate variability, cognition, stress

## Abstract

Contemplative practices, such as meditation and yoga, are increasingly popular among the general public and as topics of research. Beneficial effects associated with these practices have been found on physical health, mental health and cognitive performance. However, studies and theories that clarify the underlying mechanisms are lacking or scarce. This theoretical review aims to address and compensate this scarcity. We will show that various contemplative activities have in common that breathing is regulated or attentively guided. This respiratory discipline in turn could parsimoniously explain the physical and mental benefits of contemplative activities through changes in autonomic balance. We propose a neurophysiological model that explains how these specific respiration styles could operate, by phasically and tonically stimulating the vagal nerve: respiratory vagal nerve stimulation (rVNS). The vagal nerve, as a proponent of the parasympathetic nervous system (PNS), is the prime candidate in explaining the effects of contemplative practices on health, mental health and cognition. We will discuss implications and limitations of our model.

## Introduction

**Figure 1:**
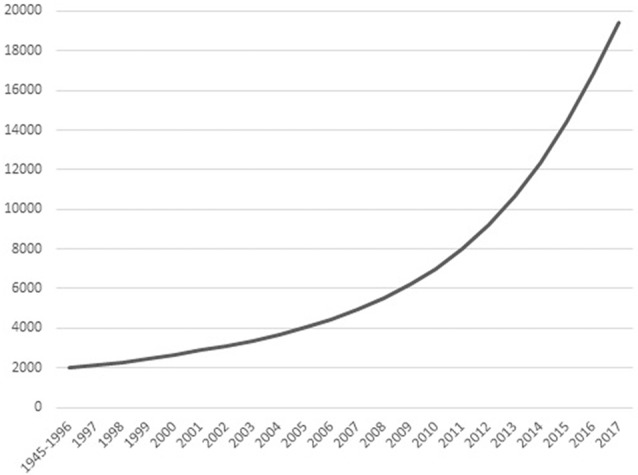
Cumulative number of scientific publications on contemplative activity (ContAct), from the 1945–1996 bracket to 2017 per individual year. Obtained from Web of Science in January 2018 using the search terms: “mindfulness” OR “meditation” OR “yoga” OR “tai chi.”

The past 50 years have shown an increasing interest in eastern contemplative traditions in Europe and North-America. These traditions include meditation styles and mindfulness, as well as mind-body exercises like tai chi chuan (TCC) and yoga. What most of these practices have in common is not only their origin in eastern philosophy and religion, but also the goal to enhance individual physical and mental health. Scientific research has followed the popularity of contemplative activities (ContActs). Figure 1 shows that the cumulative number of relevant publications since 1945 follows a quadratic pattern, with total number of publications within a decade increasing from 2,412 between 1997–2006 to 12,395 between 2007–2016 (Web of Science, February 2018). The number of clinical trials on meditation, mindfulness, yoga, TCC or qi gong each year alone increased from a little under 20 in the year 2000 to about 250 in 2014, citations in the same timeframe going up from 20 in 2000 to 7,112 in 2014 (Web of Science, February 2018).

The current article reviews scientific insights in potential health benefits. In particular, we investigate the role of particular breathing techniques (low respiration rate, long exhalations) integral to contemplative activities and show that these techniques are prime candidates in explaining the benefits of ContActs for health and mental health. Furthermore, we provide mechanisms and a neurophysiological model that can explain how respiratory patterns produce these effects; through vagal nerve stimulation.

Studies on contemplative practices have reported a plethora of positive effects on health, mental health and cognition (for reviews, see Shapiro et al., 2003; Grossman et al., 2004; Ospina et al., 2007; Wahbeh et al., 2008; Büssing et al., 2012; Lee and Ernst, 2012; Forbes et al., 2013). However, not much literature has been devoted to revealing the mechanism underlying reported benefits. The current article is intended to fill this gap. Hereby, we hope to give extra incentive for research on the mechanisms of ContAct action and inform traditional practices, thereby giving opportunity to innovate styles with new exercises and targeted interventions.

## Contemplative Traditions

For the purpose of this article we define contemplative activities as activities that involve conscious and attentive exercise aimed at changing one’s mental state, contemplation in meaning comparable to “praying” and “meditating.” We have deliberately not chosen the concept mindfulness because it is associated with particular practices, instructions and states, as we will discuss later. Despite the similarities between ContActs reflected in the definition, a few differences among ContActs are worth explaining here, because they are also used to position interventions in research.

The most common, the most referenced, the most studied and largest subgroup of ContActs is *meditation*. Most meditation traditions come from east and south Asia and are originally Buddhist or Hindu in nature. Zen Buddhist meditation (originating in China from a marriage of Buddhism and Taoism), loving-kindness meditation (Tibet), vipassana (India) and transcendental meditation (India) are popular styles. Yet there are also European and Middle-Eastern forms such as acem from Norway, Christian monastic traditions (Egan, 1991; Studzinski, 2009) and Sufi Islamic meditation: muraqaba and whirling (Cakmak et al., 2011, 2017; Nizamie et al., 2013).

In an attempt to classify meditation traditions according to their differentiated instructions, a distinction has been proposed between two types: *focused attention* meditation (FA) and *open monitoring* meditation (OM, Lutz et al., 2008; Lippelt et al., 2014). FA instructions emphasize attention to a particular focus, almost always the breath, along with means how to handle distractions and refocus attention. Zen meditation is commonly seen as an archetypical form of FA. OM stresses the spreading of attention on multiple endogenic and exogenic stimuli, having fleeting awareness of multiple sensory modalities, emotions and thought. Vipassana is popularly regarded as an exemplar of this style. There is no strict separation between the two types in zen and vipassana, and most traditions blend one style into the other. Overall, OM is frequently seen as a more advanced level exercise than FA, and is thus practiced more by experts as compared to novices.

In a further categorization attempt by Lutz and colleagues (Dahl et al., 2015; for another three-dimensional classification, see Lutz et al., 2015), more meditation styles are classified based on their most emphasized and practiced techniques. The resulting framework has three meditation families: the attentional, the constructive and the deconstructive. Both FA and OM styles belong to the attentional family. The constructive meditations are aimed at improving the well-being of oneself and others, exemplified by compassion and loving-kindness mediation. The deconstructive practices focus on breaking habits of perception, affect, thought and behavior: most *mindfulness meditation* falls in this category. Note however, that the term mindfulness has been used in a different meaning as well. It refers not only to a category of meditative practice; it can also refer to a mental state, or even the ultimate enlightenment goal of these practices. As a mental state, mindfulness refers to a state of *meta-awareness*, in which the practitioner observes emerging feelings and thoughts without judgment (*non-judgmentality*). The state of mindfulness can actually be the target of both FA and OM exercises.

Another subgroup within ContAct, here referred to as *mind-body exercises*, is more multi-modal. It involves both meditation and physical exercise; such as stances, positions, complex movements and muscle relaxation techniques. Common traditions are the many styles of Indian yoga and Chinese styles like TCC and qi gong. Origins of these styles differ, but most also have religious or mystical roots like meditation traditions, even when developed as a martial art (e.g., Taoism in TCC).

## The Positive Effects of ConTact

Research has found a vast array of beneficial effects in the three domains of physical health, mental health and cognitive performance.

### Physical Health

#### Cardiopulmonary Effects

Multiple reviews on different ContActs report a decrease in *cardiometabolic risk factors* and an increase in *cardiopulmonary health* and *fitness*, according to a meta-analysis this is most consistently reflected in lowered heart rate, blood pressure and blood lipid profile across practices (Ospina et al., 2007). Reviews on separate practices confirm this for meditation styles such as mindfulness meditation and transcendental meditation (Grossman et al., 2004; Walton et al., 2004), as well as the mind-body exercises yoga (Büssing et al., 2012; Posadzki et al., 2014) and TCC (Jahnke et al., 2010; Lan et al., 2013), where one meta-analysis indicates increased erobic capacity as well (Taylor-Piliae and Froelicher, 2004). However, Cochrane reviews on transcendental meditation, TCC and qi gong state that even though there is suggestion to their positive effect, definitive conclusions on their efficacy cannot be drawn because of a lack of high-quality long-term trials (Hartley et al., 2014a,b, 2015).

#### Anti-Inflammation

ContAct reviews also report *immunological improvements*; most studies find functional *anti-inflammatory effects*, where meta-analyses indicate that the most commonly reported decreases of pro-inflammatory markers are in C-reactive protein and pro-inflammatory cytokines, such as tumor necrosis factor-a (Morgan et al., 2014; Bower and Irwin, 2016). Again, this enhancement can be seen with different ContActs: loving-kindness meditation (Hofmann et al., 2011), yoga (Black et al., 2013) and TCC (Jahnke et al., 2010; Lan et al., 2013).

#### Physical Function

Like other physical exercises, mind-body exercises improve *general physical function*, most notably bone density, balance, strength and flexibility (Jahnke et al., 2010; Büssing et al., 2012). Mindfulness-based stress reduction, yoga and TCC seem to ameliorate *(chronic) pain* conditions, as indicated by pain scales in conditions such as: migraine, fibromyalgia and osteoarthritis (Grossman et al., 2004; Wahbeh et al., 2008; Büssing et al., 2012). As mindfulness-based stress reduction also includes yoga-like exercises, these results can be reserved to mind-body exercises and might best be interpreted as coming from the physical exercise part of these programs, as exercise-induced analgesia is well-established (Koltyn et al., 2014) and is even comparable to medication in chronic pain conditions according to a review of multiple Cochrane reviews (Geneen et al., 2017).

### Mental Health

#### Stress Relief

Reviewed ContActs *decrease stress* and* negative affect*, and in parallel *increase well-being* and* self-efficacy*, as indicated by stress and (trait) anxiety rating scales and quality of life questionnaires (Grossman et al., 2004; Kirkwood et al., 2005; Jahnke et al., 2010; Wang et al., 2010a; Keng et al., 2011; Eberth and Sedlmeier, 2012). Furthermore, a recent meta-analysis that collapsed studies using different meditation interventions, such as FA and OM, showed that they reduce multiple physiological stress markers across styles: heart rate, blood pressure, cortisol levels and inflammatory bodies (Pascoe et al., 2017).

#### Stress-Related Psychopathology

ContActs, and notably mindfulness-based cognitive therapy, *reduce symptoms in affective psychopathology* (Chiesa and Serretti, 2011; Kuyken et al., 2015). Most reviews and meta-analyses report decrease in symptoms of depression, anxiety disorders and post-traumatic stress disorder, as measured by structured clinical interview and common clinical scales, such as the Beck Depression Inventory (Klainin-Yobas et al., 2012; Balasubramaniam et al., 2013; Chi et al., 2013; Cramer et al., 2013; Kim et al., 2013; Wang et al., 2013).

### Cognitive Performance

#### Cognitive Control

Some studies show that ContActs enhance *executive functioning* and *working memory* or act as a buffer against age-related decline of executive functions and working memory: this applies to mindfulness meditation (Zeidan et al., 2010; Gard et al., 2013), yoga (Gothe and Mcauley, 2015; Luu and Hall, 2016) and TCC (Wu et al., 2013; Wayne et al., 2014; Zheng et al., 2015). Most of the reported evidence for ContActs boosting executive functioning comes from cognitive inhibition tasks, such as the Stroop and flanker tasks, whereas the support for working memory improvement comes from span and n-back tasks. Furthermore, cognitive control states can be acutely affected through very short ContAct interventions (Colzato et al., 2012, 2015a,b; Gothe et al., 2013), although these effects are typically smaller and less robust than following prolonged ContAct practice.

#### Attentional Control

ContActs also seem to have specific effects on *attentional control* and have been reported for FA and OM (Shapiro et al., 2003; van Vugt and Slagter, 2014; Colzato et al., 2015a), mindfulness meditation (Chiesa and Serretti, 2010; Eberth and Sedlmeier, 2012) and yoga (Gothe and Mcauley, 2015), with most studies reporting enhancement effects on attentional network task components and the attentional blink. These effects are differentiable according to specific practices and can be in opposite directions (Slagter et al., 2011; Hommel and Colzato, 2017). For example, practices high in FA are associated with better sustained attention, and those emphasizing OM support flexibility in allocation of attentional resources (Lutz et al., 2009; van Vugt and Slagter, 2014; Colzato et al., 2015a). Jha et al. (2007) used the attentional network test (Fan et al., 2005) to compare FA with OM in terms of attentional subcomponents as distinguished by Posner and Petersen (1990). Jha et al. (2007) showed that FA has its effects on the alerting component (detecting a stimulus) and OM on the orienting component (allocating attentional resources). Surprisingly, they found no differences on performance monitoring. Andreu et al. (2017), however, did find acutely enhanced performance monitoring by meditation. In people diagnosed with attention deficit hyperactivity disorder, meditation and mind-body exercises actually enhanced attentional functioning (Rubia, 2009; Mitchell et al., 2015; Herbert and Esparham, 2017), although an earlier Cochrane review was unable to draw conclusions due to a lack of clinical trials (Krisanaprakornkit et al., 2010).

#### Global Cognition and Creativity

Attention and control are not the only psychological outcome variables of ContAct research. Shapiro et al. (2003) observed enhanced creativity through transcendental meditation, while the meta-analysis of Ospina et al. (2007) indicated increased verbal creativity as the most reliable cognitive outcome of diverse ContActs. However, these acute effects can be in different and even opposing directions, as shown by a study on convergent and divergent thinking that used short FA and OM interventions (Colzato et al., 2012). There is also evidence that *global cognitive functioning*, as measured by the mini-mental state examination or activities of daily living questionnaire is positively influenced by ContAct in aging populations with mild cognitive impairment or dementia. Studied ContActs include mindfulness meditation (Eberth and Sedlmeier, 2012) and yoga and TCC (Wu et al., 2013), as evidenced by a Cochrane review (Forbes et al., 2013).

## Effective Factors in ConTActs

As a first step towards explaining the highly overlapping effects of diverse ContActs on physical, mental and cognitive health, a straightforward approach is to analyze what they have in common. Three of the common factors we distinguish were also proposed by Hölzel et al. (2011b) for mindfulness mediation, but we formulate them in terms of activities rather than goals and propose three additional factors: *attention training*, *affect training*, *metacognitive adjustment*, *body awareness training*, *physical exercise* and the central addition: *breathing techniques*. The first three can be seen as forms of mental training and the last three as more embodied cognitive exercises. We will cover the first five factors and other proposed models before we introduce the breathing exercises.

### Attention Training

The focus of attention in ContAct practices can involve many sensory or cognitive modalities; any of the external senses, the body, the breath, thoughts or feelings. Although explicit attentional training might be absent from some mind-body exercises, many ContActs are aimed at sustaining attention, handling distraction and refocusing or shifting and spreading of attention. These attentional techniques can explain the frequently reported effects on attention and perhaps some in the cognitive control domain. However, as stated earlier, these effects can be differentiated and in opposite directions according to specific instructions, showing increased sustained attention and decreased attentional flexibility by one manipulation (FA), and showing opposed functional differences by another (OM). Attention training in ContAct might thus be better described as resulting in a shift towards either more or less (attentional) control than as a unidirectional change. Both directions of change can be adaptive to the practitioner’s intention because such metacontrol shifts result in either more persistence or more flexibility in thought (Hommel, 2015; Hommel and Wiers, 2017). A limitation to the effective strength of attention training is that transfer of effects to other contexts and untrained skills is known to be rather limited (Seitz and Watanabe, 2005; Green and Bavelier, 2012; Keshavan et al., 2014; Simons et al., 2016).

### Affect Training

Exercises we define as affect training are aimed at removing or transforming negative emotions or moods. These start with becoming aware and paying attention to negative feelings or thoughts. This is comparable to *exposure* therapy (Hölzel et al., 2011b). Subsequent instructions serve to modify the mental state. By *decentering* the meditator attempts to distance the self from the (negative) thought or feeling, trying to observe it as just a fleeting and subjective sensation, instead of a feeling that is taken personal, in effect trying to detach the observer from the observed (Bernstein et al., 2015). Associated with decentering is the attempt to treat thoughts and feelings as not necessarily representing an objective reality. This is known as *dereification*. Finally, the detachment that results from decentering and dereification helps the meditator to avoid judgment about invasive and recurring thoughts, feelings or external events, such as surrounding noise. This *non-judgmentality* is also explicitly instructed. Taking these three mental exercises together, it is easy to maintain that they can help to *reappraise* negative feelings (Hölzel et al., 2011b). Therefore, affect training, as comprising both exposure and reappraisal, could explain findings in the area of mental health, and possibly by extension, through (chronic) stress reduction: immune function and cardiovascular health.

### Metacognitive Adjustment

Both the decentering and dereification techniques belong to the domain of meta-awareness and metacognition: being aware of awareness, thinking about thinking (Flavell, 1976). By metacognitive adjustment practitioners try to change the way they process information. Many thoughts and perceptions follow a default processing route, resulting in a default interpretation and categorization of what is perceived. ContActs that involve *thought monitoring* try to identify and deconstruct fixed thought pattern, thereby deviating from this default processing route. This effect may transfer to daily life in the form of a tolerance for ambiguity and an increase in mental flexibility. As such, some forms of ContAct can be seen as executive function training, possibly transferring to situations where overruling pre-potent responses, ignoring irrelevant information, switching between tasks and rules, or keeping working memory up to date is relevant. Therefore, metacognitive strategies can explain beneficial effects of ContAct in the cognitive control domain. However, even apparently very similar cognitive training or gaming paradigms show very little transfer of training attentional control or working memory (Green and Bavelier, 2012; Melby-Lervåg and Hulme, 2013).

### Body Awareness Training

Exercises instructing for attention to different parts of the body, mostly to the skin and muscles, but also the viscera, make up the *body awareness* *training* factor. These could be mistaken as being part of the attention training factor. However, body perception is also uniquely central in affective processing and the sense of self (Damasio, 2003; Ochsner et al., 2004; Araujo et al., 2013). Some researchers even theorize that body awareness is central in the cultivation of empathy (Grossman, 2014). This makes us treat body awareness as a factor on its own. Kerr and colleagues (Kerr et al., 2008, 2011, 2013; Kemp and Quintana, 2013) have shown that interoception can be enhanced in practitioners of mindfulness meditation and TCC: tactile acuity goes up, and activity in related somatosensory and visceral cortical areas (S1, insula, anterior cingulate cortex (ACC)) shows a pattern of increased attention to specific body parts on instruction and filtering of irrelevant somatosensory information. They state that metacognition and cognitive enhancement starts in the body: somatosensory exercises are in their view early versions of the techniques involved in attention, meta-awareness and metacognition. This being the case, body awareness could be involved in producing effects on emotional and cognitive levels.

### Physical Exercise

One could argue that *physical exercise* is the most likely candidate for broad enhancement. Many studies provide evidence that physical exercise of different kinds (erobic, endurance, strength) is a strong cognitive control enhancer, resulting in better cognitive and response inhibition, and lower dual-task costs, though reviews on working memory performance are mixed (Colcombe and Kramer, 2003; Smith et al., 2011; Roig et al., 2013; Voelcker-Rehage and Niemann, 2013; Voss et al., 2013; Berryman et al., 2014; Wong et al., 2015). Exercise is also generally accepted as a cardiovascular health booster (Di Francescomarino et al., 2009; Heran et al., 2011; Korsager Larsen and Matchkov, 2016). However, the evidence for the supposed therapeutic effect of physical exercise on depression, anxiety and other stress-related conditions has been sparse, as indicated by a Cochrane review (Mead et al., 2009), despite high expectations and invested resources (Salmon, 2001). Most importantly, only a small minority of the reported ContActs provide any erobic or endurance exercise quality: the mind-body exercises, and perhaps to a smaller degree the mindfulness-based clinical programs (i.e., mindfulness-based stress reduction). This seems to rule it out as the prime candidate.

### Theories and Models of ContAct Efficacy

There is a large gap between the amount of research done on ContAct and the number and amount of detail of models proposed to explain the benefits (Schmalzl et al., 2015). (Neuro) cognitive models that have been put forward so far attribute the benefits of meditation to top-down factors such as *attention* and *metacognition*. For example, Vago and Silbersweig (2012) emphasized the role of the self in the effectiveness of mindfulness meditation, whereas Sperduti et al. (2012) highlighted the role of executive functions in all branches of meditation.

Models that describe the benefits of mind-body exercises (Wayne and Kaptchuk, 2008; Gard et al., 2014; Clark et al., 2015) incorporate *movement*, *mindfulness* and *attention*. One refers to TCC as “meditative movement,” clearly naming the two aspects of physical and mental training (Larkey et al., 2009). However, despite labeling movement as a functional component, none of these models handle physical exercise as a full factor on its own. In the component “movement,” exercise is reduced to motor coordination and skill learning, or the training of physical strength. Even though TCC is classified as mildly erobic (Chang et al., 2010), the benefits of its erobic aspect are neglected. This is peculiar in light of the extensive support for the contribution of erobic exercise to physical health, mental health and cognitive performance.

### Breathing Techniques

Two of the mind-body models also incorporate a factor that has been conspicuously absent from other models: *breathing techniques* (Wayne and Kaptchuk, 2008; Gard et al., 2014). In both of these accounts, one on yoga and the other on TCC, the breathing type described as effective is slow, deep and diaphragmatic.

#### Effects of Respiration in Theory and Research

The *breathing techniques* used in ContAct include, but are not restricted to, slowing down respiration cycles, shifting to longer exhalations compared to inhalations, shifting the main locus of respiration from the thorax to the abdomen (diaphragmatic breathing), or paying attention to “natural” breathing. Especially slow and deep breathing with emphasis on long exhalation is dominant across traditions, including zen and vipassana—though there are a few practices stimulating faster respiration patterns (i.e., the yoga technique “breath of fire”). In the physically active mind-body exercises respiration can be synchronized with movement techniques; moving with the breath. For example, in some TCC styles moving towards the body is performed on inhale and moving outward on exhale. Note that in yoga, qi gong and TCC moving is performed slowly, and thus so is the breathing cycle.

Although the word breathing is frequently mentioned in the scientific literature on ContAct, this is almost exclusively done in a purely descriptive and not an explanatory fashion. Indeed, research on breathing as a ContAct mechanism is sparse, though there are concrete physiological grounds to look at breathing as an effective factor (Brown and Gerbarg, 2005). As far as we know there are only a few articles looking at respiration in the context of ContActs directly, varying wildly in aims and measures. Danucalov et al. (2008) found increases in metabolism and oxygen uptake during yogic breathing exercises (*pranayamas*) in experts, as compared to rest and meditation conditions in a within-subjects design. The breathing exercises used included holding the breath and extending exhalation. A similar slow breathing pranayama was used by Pramanik et al. (2009) showing reduced blood pressure and heart rate at post-measure. Brown and Gerbarg (2009) reviewed their own studies on the psychophysiological effects of various breathing techniques used in Sudarshan Kriya Yoga and reported a general tendency among the breathing exercises towards relaxation: activating the parasympathetic nervous system (PNS) and deactivating the sympathetic nervous system (SNS). Cysarz and Büssing (2005) observed increased cardiorespiratory synchronization with a slow breathing zen meditation intervention in naïve subjects. Though there is clearly a lack of studies on breathing in ContAct practice, fundamental neurophysiological studies on respiration mechanics and styles do abound.

In several studies respiration types have been manipulated in an attempt to influence autonomic nervous system functioning. A study of the effect of diaphragmatic relative to normal breathing on metabolism among male cyclists, before and after a meal found reductions in heart rate and glycemia, and increases in insulin (Martarelli et al., 2011). Bernardi et al. (2001) induced hypoxia in participants and found that slow breathing exercises not only increased blood oxygenation, but also down-regulated SNS activity. Similar results are reported by Critchley et al. (2015) study on hypoxia. Many other studies show that slow and diaphragmatic breathing increases PNS activity, as measured by blood pressure, heart rate or heart rate variability (HRV; Hirsch and Bishop, 1981; Lee et al., 2003; Pal et al., 2004; Lehrer and Gevirtz, 2014; Van Diest et al., 2014; Mortola et al., 2015; Perciavalle et al., 2017; Tavares et al., 2017; for some conflicting results see and Montgomery, 1994; Conrad et al., 2007). In sum, experimental slowing of respiration seems to shift the balance between SNS and PNS activity towards the latter. Next, in light of these findings, we will look at the part of the nervous system responsible for such a shift: the vagus nerve (VN) and measures of its tone (i.e., HRV).

## The Vagus Nerve and Heart Rate Variability

### Vagus Nerve

The autonomic nervous system (Langley, 1903) is a dual-system divided in the SNS and PNS with mutual inhibitory connections, though the dual innervation can also work complementary in organs such as the heart (Jänig et al., 1983; McCorry, 2007). The SNS is responsible for the fight/flight mode of organisms. It raises heart rate, blood pressure and indirectly respiration rate. It dampens currently irrelevant homeostatic processes, but stimulates immediate availability of energy. The PNS acts as an opposing force. It is the rest/digest system of the organism. It lowers heart rate, respiration rate and increases digestion. The VN is the main affector and effector of the PNS.

The VN is a cranial nerve complex with widespread afferents and efferents on glands and visceral organs (Berthoud and Neuhuber, 2000), consists of approximately 20% efferent and 80% afferent fibers (Agostoni et al., 1957) and has many independently operating functions (Chang et al., 2015). Overall it is well-suited for relaying relaxation from the central nervous system (CNS) to the body and checking the arousal and homeostatic state of the viscera. VN activity is modulated by respiration. It is suppressed during inhalation and facilitated during exhalation and slow respiration cycles (Chang et al., 2015). Efferent and afferent VN functions overlap with the functional effects associated with ContAct practice. Therefore, the breathing exercise component of ContAct is a prime candidate mechanism behind the beneficial effects found on mental and physical health.

#### Cardiopulmonary Control

Efferent VN fibers innervate the heart and the lungs. The pulmonary efferents regulate airway size and thus volume, they lower respiration rate and indirectly endocrine secretion (Yuan and Silberstein, 2016a). Exhalation is under direct control of VN (Chang et al., 2015), whereas VN activity is attenuated during inhalation (Eckberg and Eckberg, 1982; Canning, 2006). The vagal cardiac outputs to the sinoatrial node causes slowing of heart rate, whereas SNS innervation is responsible for heart rate increase. The SNS cardiac effector is under tonic inhibition of VN, indicating indirect control on heart rate increase (Olshansky et al., 2008).

#### Anti-Inflammation

There is evidence that VN also influences physical health by suppressing inflammation. An anti-inflammatory reflex, known as the cholinergic anti-inflammatory pathway has been put forward from findings in animal studies on rats (Tracey, 2002, 2007; Pavlov and Tracey, 2015). This response is thought to inhibit a cascade of inflammatory activity and is triggered by vagal afferents monitoring immune status. However, an alternative sympathetic anti-inflammatory reflex has been proposed, explaining conflicting results in rat studies: the splanchnic anti-inflammatory pathway, where VN plays an afferent role at most (Bratton et al., 2012; Martelli et al., 2014a,c, 2016). A complete discussion of these competing pathways lies outside the scope of this review. Suffice it to say that the VN seems to be involved in anti-inflammation in humans: studies using VN stimulation paradigms report anti-inflammatory effects as well (Browning et al., 2017; Johnson and Wilson, 2018). Furthermore, after vagotomy inflammatory activity goes up. This resembles the earlier mentioned tonic inhibition on heart rate (Borovikova et al., 2000). This merits the proposition that VN mediates effects of ContAct on immunological health, specifically anti-inflammatory, and potentially those on auto-immune diseases.

#### System State Monitoring

VN afferents reach the medulla from the heart, airways, liver and gastrointestinal track. It monitors cardiorespiratory, endocrinal and immune parameters (Berthoud and Neuhuber, 2000). Mechanoreceptors in the airways signal on airway size and thus on respiration cycle and style (Undem and Carr, 2001; Canning, 2006). VN afferents on the adrenal glands relay information on the release of stress hormones, such as epinephrine and the glucocorticoids (Coupland et al., 1989; Niijima, 1992; Kessler et al., 2012). The afferent branch of the VN constantly send up homeostatic parameters to the CNS, monitoring the state of the visceral system. This branch has been characterized as the “great wandering protector” (Andrews and Lawes, 1992).

Clearly, these functions all move the system towards the rest/digest mode of operation and away from fight-or-flight. Not only does VN control heart rate and slow deep breathing, slow respiration rates with extended exhalation could also activate the PNS by VN afferent function in the airways. This is a form of respiratory biofeedback. Slow breathing techniques with long exhalation will signal a state of relaxation by VN, resulting in more VN activity and further relaxation. Though VN involvement can explain effects on health and mental health, the link with cognition is less clear. One of the links between respiration and cognition is HRV.

### HRV

#### Vagal Tone

HRV, the fluctuations in beat-to-beat intervals of the heart, has been related to VN and some measures are believed to reflect “vagal tone.” As only the VN cardiac output and not the sympathetic innervation would be able to produce rapid changes in heart rate. HRV is used as an indicator for individual physical conditioning, general health, reactivity to and recovery from high stress levels. Higher HRV is related to lower stress levels, better health and disease outcomes (Thayer et al., 2012). A frequently used HRV metric to assess vagal tone is the respiratory sinus arrhythmia, by some maintained to be the best reflection of vagal tone (Porges, 2001, 2007). This refers to the acceleration of heart rate during inhalation and deceleration during exhalation.

HRV can either be obtained in the time or the frequency domain (Task Force, 1996; for a recent review of HRV methods see: Laborde et al., 2017). High frequency HRV (HF), also referred to as respiratory sinus arrhythmia, is seen as a measure of vagal tone, whereas low frequency HRV (LF) is thought to represent sympathetic activity. The ratio between the two (LF/HF) represents autonomic balance, where a smaller number indicates vagal dominance. However, studies have shown that vagal activity is also reflected in LF and furthermore that LF does not reflect the SNS (Martelli et al., 2014b), making the ratio unusable as an indicator of autonomic balance. Currently, most studies confirm that specific measures in the time domain (e.g., root mean square of successive differences, peak-valley method) best reflect vagal tone (Penttilä et al., 2001), though some studies state that HRV, notably respiratory sinus arrhythmia, is not a reliable indicator of vagal tone at all (Grossman and Taylor, 2007). Individual HRV varies widely through time and during various activities, such as physical exercise (Hottenrott et al., 2006). Three types of measurements in time can be defined: resting or baseline HRV, reactivity HRV and recovery HRV (Laborde et al., 2017). Resting HRV is obtained with the participant sitting down, not performing any specific activity and can be seen as an individual’s baseline level. Reactivity HRV is obtained during an activity or intervention, such as a cognitive task or a breathing exercise. This short-term HRV tends to drop during a cognitive challenge (Wood et al., 2002). Recovery HRV refers to the return to baseline afterwards. In this article, when we mention HRV we refer to resting state HRV measures that best reflect vagal tone (HF and aforementioned time-domain measures), unless stated otherwise. Changes in these measures thus reflect changes in tonic vagal tone. An exception consists of most of the reported studies on respiration patterns: in this case the HRV concerns reactive HRV and in some cases recovery, and thus phasic changes in vagal tone.

As stated earlier, HRV is regarded as an indicator of physical, but also cognitive health. Indeed, there is a concrete link between HRV and cognition, first sketched in the neurovisceral integration model of Thayer and Lane (2000).

#### HRV and Cognition

The neurovisceral integration model (Thayer and Lane, 2000; Thayer, 2007; Thayer et al., 2009) posits bi-directional cortical influences on autonomic functioning and integrates CNS and autonomic functioning. It builds on the work of Benarroch (1993, 1997) on the Central Autonomic Network (CAN), a network of brain areas for goal-directed behavior involved in modulating the viscera. These areas are mostly limbic and include the insula, ACC, amygdala and hippocampus. The neurovisceral integration model extends this to prefrontal structures (orbitofrontal, medial and lateral PFC). These regions are able to influence HRV and initiate endocrine responses through the VN. But the integration of CNS and autonomic nervous system also works bottom-up: projections from VN afferent medular termini reach limbic and cortical regions, affecting cognitive control. This framework provides a basis for a connection between executive functions on the one hand, and body relaxation on the other. Indeed, studies by Thayer and others show evidence of a positive association between HRV and PFC activity and subsequent improvements in executive functions, notably cognitive inhibition. PFC seems to exert tonic inhibition on heart rate (and the amygdala), and greater activity of the PFC is associated with higher HRV (Lane et al., 2001, 2009). Hansen et al. (2003, 2004) provided further evidence of this relation in individual differences studies: higher HRV is associated with better executive functions and working memory performance. In the view of Thayer and colleagues, HRV can be seen as a peripheral index of the adaptability of the nervous system and thus the organism. HRV increases with goal-directed behavior and emotion regulation, and reduced HRV is indicative of cognitive stress. Clearly, the CAN and these experimental findings give grounds for explaining executive function enhancement following ContActs as originating from VN, through upward projections producing functional and structural changes in the executive network.

In a recent update of the neurovisceral integration model (Smith et al., 2017), which adopts a hierarchical network architecture, the relative weight of top-down and bottom-up influences can be adjusted. This leaves room for learning or the training of autonomic responses. For example, a non-adaptive dysfunctional stress response can be modulated or go extinct by the reappraisal of threat (top-down) or exposure to the stressor (bottom-up). This means that stress levels could be downregulated by lower level state feedback that is associated with unthreatening situations. In our account these are the pulmonary parameters: low respiration rate and long exhalation.

### Vagal Nerve Stimulation

The many functions of VN have led researchers and clinicians to develop electrical or behavioral intervention techniques for VN stimulation (VNS). These techniques are promising for clinical application and for improving cognitive performance. At the same time, the pattern of results observed following VNS mirror those obtained by ContAct, making VN involvement likely, and thus breathing exercises a promising candidate for stimulation.

#### Electrical VNS

Electrical VNS (Henry, 2002; Groves and Brown, 2005; Yuan and Silberstein, 2016b) was originally used to treat epilepsy. However, because it also increased the mood of stimulated patients it found its way as an approved therapy for depression (Johnson and Wilson, 2018), especially treatment resistant depression (Müller et al., 2018). It is also used to treat cardiovascular disease (Das, 2011; Johnson and Wilson, 2018) and as mentioned earlier, VNS has also shown acute anti-inflammatory effects (Browning et al., 2017; Johnson and Wilson, 2018), possibly through the anti-inflammatory pathway (Borovikova et al., 2000; Yuan and Silberstein, 2016c).

VNS is also applied to respiratory conditions. A study on guinea pigs has shown that strength of stimulation makes a difference: high voltage produces the predicted VN effects bronchoconstriction, reduced heart rate and blood pressure, while low voltage only produces the pulmonary effects (Hoffmann et al., 2012). Studies in humans, in contrast, show that VNS can actually produce airway relaxation in asthma patients during acute episodes, as indicated by an increase in forced expiratory volume (Miner et al., 2012; Steyn et al., 2013). In other words: stimulating afferent branches of VN during exacerbations (shortness of breath) produces longer exhalations and therefore slowing of respiration rate.

VNS has been shown to affect cognitive functioning, for example memory consolidation and recognition (Clark et al., 1999; Ghacibeh et al., 2006; Vonck et al., 2014). Effects found on mood and memory can be interpreted through the vagal projections into the central autonomous network. It is also supposed that by vagal projections to the locus ceruleus, norepinephrine levels are influenced in midbrain and forebrain structures. This proposition is paradoxical as norepinephrine increase is more associated with sympathetic than with parasympathetic activity, and indeed evidence for norepinephrine release by VNS is mixed (Ventura-Bort et al., 2018). We propose that VNS actually increases PNS activity and that norepinephrine projections play a minor role, as shown by recent neuroimaging studies (Frangos et al., 2015). Clearly, VNS not only shows effects on well-documented afferent and efferent functions of VN, but also fits with the neurovisceral and CAN account of cortical VN projections.

#### Transcutaneous VNS

Transcutaneous VNS (tVNS) is a new non-invasive tool that is used to electrically excite the auricular or cervical branches (afferent) through electrodes placed on the ear or neck. Though this line of research is in its infancy, preliminary results also show an association between tVNS and VN-related afferent functions and projections. Shiozawa et al. (2014) concluded from a review of neuropsychiatric studies that tVNS can reduce symptoms of depression. Furthermore, a recent study has shown that (cervical) tVNS indeed shifts autonomic balance from the SNS to the PNS in tinnitus patients, as indicated by the increase of multiple vagal tone HRV measures (Ylikoski et al., 2017). Neuroimaging studies have also shown that cortical and sub-cortical regions identified in CAN are activated during both cervical and auricular tVNS (Dietrich et al., 2008; Frangos et al., 2015).

Few studies have been conducted using tVNS to influence cognitive behavioral performance. However, two studies have shown phasic changes in associative memory (Jacobs et al., 2015) and in response selection (Steenbergen et al., 2015) following tVNS. Interestingly, tVNS also causes effects that would be expected if VN efferent function would be modulated, by increases in vagal tone. Multiple studies and reviews show an increase of PNS activity, and some also show a decrease of SNS activity (Popov et al., 2013; Clancy et al., 2014; Murray et al., 2016; Zhou et al., 2016). Furthermore, tVNS is also associated with anti-inflammatory effects (Wang et al., 2014, 2015). These results overlap strongly with those obtained in ContAct studies.

#### Behavioral VNS

There are also behavioral forms of VNS (*vagal maneuvers*), which are supposed to stimulate VN bilaterally. The Valsalva technique; pinching the nose closed and then trying to exhale through the nose, is best known for returning normal pressure to the inner-ear cavities when changing altitudes (Arnold, 1999). It is initiated by flexing the abdominal muscles and extending exhalation (in clinical or laboratory setting by blowing into a balloon), showing a strong similarity with breathing techniques in ContAct. Even further, extending, slowing and holding respiration are all considered vagal maneuvers on their own, stimulating the VN. All of these vagal maneuvers have been shown to slow heart rate (bradycardia). We propose that the breathing exercises of ContAct might be seen as a form of behavioral VNS.

Overviewing the functions and applications of VN, one can see its potential as a mediator between respiratory patterns employed in ContActs and the reported effects on health, mental health and cognition. This will be further outlined in our model.

## The Respiratory Vagal Stimulation Model of Contemplative Activity

**Figure 2:**
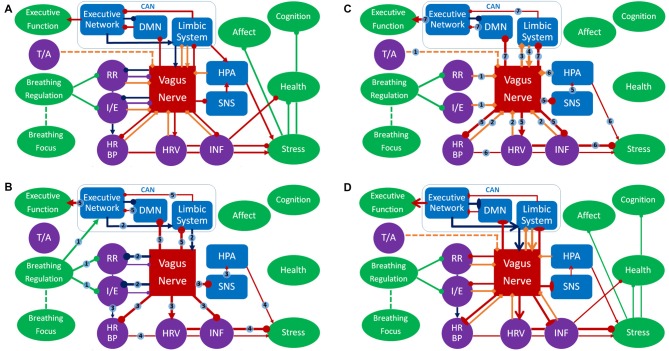
Panel **(A)** represents an overview of the respiratory vagal nerve stimulation (rVNS) model of ContAct. See the text body for more details. There are two pathways through which respiration style stimulates VN: direct and indirect (biofeedback through afferent projections), shown in **(B,C)** respectively. **(D)** The tonic changes in the networks and the long-term effects. Color coding: red = VN, blue = other anatomy, purple = physiology, green = function, dark blue = direct route, orange = indirect route. Arrow-ends represent role: triangle = activating or increasing, circle = deactivating or decreasing, bladed = structural increase, ellipsis = structural decrease, diamond = afferent. Numbers on lines represent the temporal sequence during stimulation and the thickness of lines the phasic relative synaptic weight of the connection as a result. The dashed line represents the hypothetical afferent pathway of the thoracic/abdominal ratio to VN. RR, respiration rate; I/E, inhalation/exhalation ratio; T/A, thoracic/abdominal respiration ratio; HR, heart rate; HRV, heart rate variability; INF, inflammation state; SNS, sympathetic nervous system; HPA, hypothalamic pituitary adrenal axis; CAN, central autonomous network; DMN, default mode network.

The model, as depicted in Figures 2A–D, has a number of assumptions, inductions and predictions. These can be roughly divided into: (a) ContAct breathing; (b) respiratory stimulation; and (c) long-term effects. This will be followed by a definition of terms and measures.

### ContAct Breathing

ContActs are multi-modal interventions that can incorporate many different techniques and instructions. However, one of the most prominent and common ContAct techniques is respiratory regulation, in other words: breathing exercises. These breathing exercises have in common the instructions to focus on and slow down respiration, and/or extend exhalation. In Figure 2 this is represented by the node “breathing regulation” inhibiting the nodes for respiration rate and inhalation/exhalation ratio, in other words: these exercises lower respiration rate and ratio. However, even exercises where the breath is just an attentional focus will lead to non-volitional adjustments of respiration. Practitioners just being aware of their breath enter slower and deeper respiration cycles. This can be caused by individuals’ previous experience with slow and deep breathing techniques, whose respiratory patterns will be automatically superimposed on current respiration. Another way is through the commonly slow pace of guided meditation instructions themselves: practitioners will sync their respiration to this rhythm. As focusing supposedly also leads to respiratory adjustments, similar to the breathing exercises, this is fit in the model overview (Figure 2A) by the node “breathing focus” showing a dashed line towards the node “breathing regulation.” Frequently adopting these respiration patterns (slowed and with longer exhalations) can explain a significant part of the efficacy found within ContAct practice. Though the ContActs are diverse, they have shown a similar pattern of beneficial effects on health, mental health and cognition: mostly in stress-related conditions and performance. This pattern can be explained by these controlled breathing exercises.

### Respiratory Stimulation

The main mediator of controlled breathing exercises on the described health, mental health and cognitive effects is VN. We posit that specific respiration patterns serve as respiratory VNS (rVNS). The styles of respiration providing rVNS are controlled breathing techniques that slow and deepen respiration and extend expiration (Garcia et al., 2013), and possibly those that put emphasis on relatively stronger diaphragmatic breathing. Note that rVNS is bilateral stimulation by nature, as opposed to unilateral electrical stimulation of VNS and tVNS. In Figure 2 this is represented by the nodes respiration ratio and inhalation/exhalation ratio inhibiting VN. As such, rVNS is one of the main mechanisms of ContAct efficacy. rVNS can have two routes: direct and indirect. Figure 2B represents the direct stimulation and Figure 2C the indirect pathway of rVNS, which is temporally ordered by connection numbering.

#### Direct Route

As can be seen in the dark blue path in Figure 2B, adopting a low respiration rate and small inhalation/exhalation ratio can directly stimulate VN, top-down from the executive network, by its own efferents (connections 2). This phasic increase in vagal activity increases reactive HRV, lowers heart rate and blood pressure (also by cardiorespiratory coupling), inhibits the SNS and indirectly the hypothalamic pituitary adrenal axis, and potentially activates an anti-inflammatory pathway (connections 3), resulting in a decrease of acute stress levels (connections 4). Critically, we also posit that VN activation statically increases cognitive control through CAN projections (connections 5).

#### Indirect Route

Indirectly, afferent VN pathways constantly signal respiratory patterns upwards, in this case characterizing a state of relaxation and low-threat. As a result, efferent VN activation further increases vagal tone and produces associated physiological consequences (e.g., lowering heart rate, blood pressure, increasing HRV); a loop of relaxation ensues. In Figure 2C this route is represented by orange diamond arrows (afferent) that signal on respiratory patterns, possibly including the ratio of thoracic and abdominal expansion (connections 1), and the cardiac patterns influenced by the direct route (connections 2). This relaxed body state goes up from VN to the limbic system (connections 3), that in turn re-activates VN (connections 4), increasing its excitatory weight on cardiac and inflammatory patterns of physiological acute stress (connections 5 and 6) and statically enhances cognitive control (connections 7). The indirect route can be seen as a form of biofeedback and is responsible for long-term changes in vagal tone. In this the respiratory patterns play a key role: a recent study using electroneurogram to map respiratory pattern signaling of the left VN, showed a near perfect overlap between this mapping and actual respiratory cycles (Sevcencu et al., 2018). Note that the left afferent VN is the locus of (t)VNS.

Diaphragmatic breathing might provide rVNS independent of respiration rate and inhalation/exhalation ratio, represented by the dashed inhibitory path of the thoracic-abdominal ratio to VN in Figure 2C. When oxygen demand is high during exercise or stress, SNS becomes active, and thoracic respiration goes up, and abdominal muscles are actively inhibited (Secher and Amann, 2012). When oxygen demand is low, in times of rest and digest, the vagal dominant state, the ratio shifts more towards abdominal respiration. The abdominal-thoracic respiration ratio of the rest-and-digest mode of the PNS should thus be similar to the ratio during ContAct practice. rVNS produces a wide range of effects in health, mental health and cognitive flexibility of the practicing individual, in the short as well as the long-term.

### Long-Term Effects of Respiratory Stimulation

#### Stress Reduction and Anti-Inflammation

Though rVNS produces a phasic change in PNS activity during and right after practice, in the long term it also results in a tonic shift in autonomic balance, shown in Figure 2D. As PNS activity goes up, SNS activity goes down. This shift is known as vagal dominance. In vagal dominance chronic stress and stress-related conditions are attenuated. Relaxation or rest and digest behavior increases. VN is responsible for the physiological effects of the red arrows in Figure 2D: heart rate, blood pressure and inflammatory response go down, whereas HRV goes up, which in turn also affects (chronic) stress. This works directly through tonal activity of the PNS, but also indirectly through inhibition of the SNS by VN. Specifically, reduction of the (chronic) stress response has positive effects on cardiovascular health and on stress-related psychopathology, shown by the stress node inhibiting the health and negative affect nodes, but also the general cognition node. Furthermore, vagal dominance also leads to better immune functioning and attenuation of inflammatory conditions. As can be seen in Figure 2D: VN inhibits the inflammation node, which inhibits the health node. These structural and tonic physiological changes in the networks are represented by bladed (activation) and elliptic arrows (inhibition) in Figure 2D.

#### Cognitive Performance

rVNS increases vagal dominance in both resting state and in active states demanding behavioral and cognitive flexibility. The CAN (Benarroch, 1993) is a CNS network that receives its projections from VN and overlaps with the executive functioning network. The executive functioning network is not only dependent on autonomic balance for proper functioning, but can also be functionally and structurally changed by CAN activity. Enhancement of executive functions in ContAct practice results from rVNS of CAN, by structurally changing and activating the hubs of the executive functioning network and increasing their connectivity. In Figure 2D this is represented by the bladed red arrow path going up from VN to the limbic system to the executive network and then to the executive function node. Likewise, we hypothesize that default mode network (DMN, Raichle et al., 2001) hubs and internal functional connectivity are decreased, while DMN connectivity with the executive network is increased. The role of the DMN can be visualized as being inhibited by vagal projections and having a two-way inhibitory pathway with the executive network, as represented in Figure 2. These pathways are activated both phasically (Figures 2B,C) and tonically (Figure 2D).

### Terms and Measures

1. Different forms of volitional control of respiration are defined as *controlled breathing techniques*. For this definition to be valid it should be possible for humans to put respiration under volitional control, overriding central pattern generated drive, and this has indeed proven possible for the diaphragm (Kolář et al., 2009).
2. *Vagal tone* is a construct relating to intra-individual tonal levels of PNS activity. Vagal tone can indirectly be indexed using HRV, notably respiratory sinus arrhythmia.
3. *Vagal dominance* refers to a relatively higher activity of PNS over SNS. Vagal dominance (PNS hyperactivity and SNS hypoactivity) should be observable in physiological measures of PNS (i.e., HRV) and SNS (i.e., pre-ejection period, skin conductance, cortisol) activity. Vagal dominance can be increased both in acute and chronic time settings (Porges, 2001, 2007). However, in this work it is defined as a macro-state of autonomic balance, spanning minutes and hours, not a micro-state, changing millisecond to millisecond, for example: in heart node activation.
4. *HRV* is also a suitable inverse measure of acute and chronic (psychological) stress (Porges, 1992, 1995). HRV can be used as an indirect indicator of intra-individual and possibly inter-individual differences in executive functions (Thayer et al., 2009).
5. As *rVNS* is a form of VNS, results obtained from studies using other modes of VNS should resemble those from ContAct studies in similar conditions, although not necessarily with perfect overlap.
6. The *stress* *release* refers to stress responses over larger scales of time; not the acute adaptive arousal employed in challenging situations, but the perseverative and chronic kind; in other words: the default stress response (Brosschot, 2017).

### rVNS: Evidence and Possible Mechanisms

What evidence is there for respiration as a mode of VNS? In our model there are two ways respiration can stimulate VN: directly and indirectly. In the direct route, slow breathing and extended exhalation are caused by vagal activity. This follows from the previously mentioned role of VN in respiratory affective and effective processing (slowing and exhalation). Controlled breathing in this form thus uses the vagal nerve as effector and increases its activity volitionally, if only momentarily. The indirect route involves stimulation through biofeedback and follows from physiological feedback theory: by adopting physiological body patterns associated with relaxation and low threat situations (i.e., slow breathing) vagal afferents project this state to the CNS, which interprets this as a reflection of the current contextual threat level, and proceeds by further adopting a rest-and-digest state top-down, again through VN. The indirect route is responsible for more long-term tonic changes of vagal tone. By either route, breathing styles with low respiration rate and low inhalation/exhalation ratio should show increases in vagal tone, though in slightly different timeframes (Keyl et al., 2001).

#### Evidence

Most experimental studies show higher HRV following breathing instructions, consistent with the involvement of rVNS. In particular, there is ample evidence that slow and deep breathing increase HRV indices of vagal tone (Hirsch and Bishop, 1981; Pal et al., 2004; Larsen et al., 2010; Lehrer and Gevirtz, 2014; Critchley et al., 2015; Mortola et al., 2015; Tavares et al., 2017) and lowers stress markers such as: heart rate, blood pressure and salivary cortisol (Lee et al., 2003; Pramanik et al., 2009; Perciavalle et al., 2017). Van Diest et al. (2014) looked specifically at the effects of different inhalation/exhalation ratio at either slow or normal respiration rate on different HRV measures (peak-valley, HF): higher HRV (both measures) was reported in the slow respiration condition, but only for extended exhalation, inhalation/exhalation ratio: 0.24, and not for extended inhalation, inhalation/exhalation ratio: 2.33. Though normal ratios were not included, this study most clearly shows the stimulating effects of the specific respiration styles on VN. For another example of extended exhalation, albeit with a completely different aim and context: a study on native American flute playing showed significant increases in HRV during playing, contrary to what one would expect during exerting activity (Miller and Goss, 2014). It needs no mention that playing any piping instrument involves extreme extended exhalation.

As far as we know, few studies report a decrease in HRV after controlled breathing, and these are primarily found outside the domain of ContActs. Sasaki and Maruyama (2014) gave instructions to participants to “control breathing,” without emphasizing a particular style (rate or ratio), and compared that to spontaneous breathing. This resulted in lower HRV, which may be the result of an increase in mental effort, stress, and thus SNS activity. Indeed, earlier reports also show a decrease of HRV when breathing is only “controlled” (Bernardi et al., 2000) as compared to directed in a specific direction. Note here the apparent contradiction with our own view that paying attention to the breath would result in lower respiration rate and possibly smaller inhalation/exhalation ratios: instructing to “control” vs. “focus on” seems to have opposing results on autonomic balance.

As we are reviewing breathing techniques that are practiced in ContActs, studies that look into autonomic functioning through ContActs employing these kinds of techniques should report increased vagal tone. Indeed, HRV increases in almost all forms of ContAct, consistent with the rVNS hypothesis. Different forms of meditation (e.g., body scan, FA, OM acem, zen) and mind-body exercises such as yoga, all show increases in vagal tone HRV in healthy participants (Ditto et al., 2006; Phongsuphap et al., 2008; Wu and Lo, 2008; Tang et al., 2009; Markil et al., 2012; Melville et al., 2012; Nesvold et al., 2012; Telles et al., 2013). One exception is a study that involved the earlier mentioned “breath of fire” (Peng et al., 2004) that showed a decrease in HF, LF and LF/HF ratio. This is not surprising and consistent with our biofeedback perspective, as breath of fire is strictly speaking controlled hyperventilation and would thus rather result in SNS activation and PNS inhibition. Though ContActs by great majority do not employ this particular rare technique, this nonetheless stresses the importance of mapping actual practiced techniques in every ContAct to their outcomes. From these abundant, though correlational, findings on respiration and vagal tone we conclude that a form of rVNS plays a role in ContAct efficacy. However, less clear is what the exact physiological mechanisms of stimulation might be.

#### Possible Mechanisms

The first possible physiological mechanism for these respiratory patterns to stimulate VN (as biofeedback) is by way of the baroreceptor reflex (Vaschillo et al., 2002; Lehrer et al., 2003). This reflex is responsible for regulating blood pressure and is triggered by stretch-activated mechanoreceptors (baroreceptors) in blood vessels, which leads to activation of the vagal branch of the heart node, that reduces heart rate and subsequently blood pressure. The threshold for triggering this reflex (cardiovagal baroreflex sensitivity) can be lowered by a respiration rate around 0.1 Hz or about 6 breaths per minute. Interestingly, this is exactly the same respiration rate that is reported in respiration studies as having the highest increase of HRV. Lowering the sensitivity results in more frequent reflexes, lower heart rate, and increased vagal tone (Song and Lehrer, 2003; Lin et al., 2012; Wang et al., 2010b; Lehrer and Gevirtz, 2014; though see Tzeng et al., 2009; for an exception). This mechanism is a faster indirect route between respiration rate and heart rate, as mediated by VN, than the biofeedback route through VN afferent subcortical projections signaling broad relaxation.

The second possible feedback mechanism is even more direct and comes from the lungs themselves: the pulmonary mechanoreceptors. These VN afferents directly relay tidal volume upstream and are responsible for initiating particular physiological responses, notably the Hering-Breuer reflex (Breuer, 1868; Hering, 1868). The reflex is triggered by significant lung volume increase (e.g., during inhalation) and inhibits the central inflation drive, resulting in extended exhalation and slower respiration. In this way, when a practitioner starts a breathing exercise with a deep breath (a long inhalation), this immediately triggers the reflex, resulting both in activation of VN as well as the initiation of respiration styles that further relay relaxation. Furthermore, the dominant and supported view is that the mechanoreceptors, together with central pattern generated drive, are also responsible for respiratory sinus arrhythmia (Taha et al., 1995; Eckberg, 2003; Mortola et al., 2015).

The slowest of the indirect routes: biofeedback, where low respiration rate and small inhalation/exhalation ratio signal a resting state to the CNS is consistent with the James-Lange physiological feedback hypothesis of emotion and similar accounts (Levenson, 1994; Critchley and Garfinkel, 2015). The theory, independently proposed by William James and Carl Lange, maintains that the identification and experience of an emotion follows from peripheral physiological responses (e.g., arousal), instead of the other way around. The kind of emotion experienced depends on the interpretation of the physiological state and the appraisal of the context in which it is triggered. So, the physiological stress response precedes the subjective emotional experience of fear or sadness. Following this argument, bottom-up changes to dysfunctional emotional states can be produced by changing the physiological state of the body; in other words: relaxing the body relaxes the mind.

In sum, there is evidence that particular breathing exercises (with low respiration rate, small inhalation/exhalation ratio) are capable of stimulating the vagal nerve (rVNS), though the exact mechanisms of stimulation are proposed, not proven (i.e., baroreflex). The next question is how prolonged increase in vagal tone results in the beneficial effects found on health and mental health. Vagal dominance is contingent on consistent physiological relaxation. It therefore produces (chronic) stress release, and thereby prevents or ameliorates stress-related pathology and etiology.

### Relaxation vs. Stress: Health and Mental Health Outcomes

Although SNS and PNS can be simultaneously active in a particular domain, they mostly operate as opposing forces (Berntson and Cacioppo, 1999; Freeman, 2006). SNS activity goes together with PNS inactivity and* vice versa*. Therefore, PNS hyperactivity (as indicated by HRV) also reflects SNS hypoactivity: vagal dominance. Plainly stated: relaxation means absence of stress. If ContActs work through relaxation by respiratory stimulation of the PNS, then stress should go down. This explains the observation that syndromes relieved after ContAct practice are often those associated with stress and SNS dominance.

The role of (chronic) stressors on the development of cardiovascular disease, through the cardiovascular response (Obrist, 1981) of the SNS causing atherosclerosis and hypertension, is well documented (Allen and Patterson, 1995; Rozanski et al., 1999; Thayer et al., 2010). That stress influences immune function is also well-known. Initially and acutely, stress suppresses immune function, but chronically it exacerbates immune response (Sapolsky et al., 2000; Haroon et al., 2011; Ménard et al., 2017). Additionally, stress seems to worsen auto-immune disease (Elenkov and Chrousos, 2002). Furthermore, there are indications that the two systems are related in their morbidity by SNS-PNS imbalance: recovery of both cardiovascular and immunological markers is impaired after stressors, when baseline vagal tone is low (Weber et al., 2010). Also noteworthy is the existence of an inverse relationship between HRV and both inflammation and the risk of cardiovascular disease (Haensel et al., 2008). Bringing these findings together, HRV seems suitable as a multi-index of health: of physiological stress (Porges, 1995), as a measure of cardiovascular risk (Thayer et al., 2010) and of immunomodulation (Thayer and Sternberg, 2010).

In the mental health domain, mood disorders such as depression are widely recognized as being stress-related. They are often accompanied or triggered by acute or chronic life event stressors in the prodromal phase (Gold and Chrousos, 2002; Duman and Monteggia, 2006; Orosz et al., 2017). Depression has also shown a relation with the other stress-related diseases: there is a link of depression with occurrence of cardiovascular disease (Hare et al., 2014) and with the likelihood of having an overreacting immune system (Dantzer et al., 2008; Miller et al., 2009; Felger and Lotrich, 2013). All in all, these systems and their pathologies seem to be interrelated, wherein the common denominator is autonomic balance.

A healthy autonomic balance is vagally dominated and comes about by stress relief produced by PNS activation and SNS deactivation. If this is the way the aforementioned pathologies are positively affected, then there should be a clear negative relationship between vagal tone and the risk factors and symptoms of these conditions. Indeed, HRV shows a negative correlation with cardiovascular disease in children and adults (Tully et al., 2013; Oliveira et al., 2017) and even directly predicts hypertension (Schroeder et al., 2003). It has an inverse relationship with inflammation (Lampert et al., 2008; Kemp and Quintana, 2013), inflammation in depression (Carney et al., 2007), depressive symptoms in children and adults (Sgoifo et al., 2015; Koenig et al., 2016), perseverative cognition (Ottaviani et al., 2016), bipolar disorder symptomology (Faurholt-Jepsen et al., 2017), general anxiety and disorders (Cohen and Benjamin, 2006; Tully et al., 2013; Chalmers et al., 2014) and has recently even shown a negative correlation with schizophrenia (Clamor et al., 2016). Though schizophrenia is not considered a stress-related disorder, the role of HRV in schizophrenia is intriguing considering the interplay of dysfunctional emotional regulation and executive functions in its symptomology.

Stress has a negative association with executive or PFC function. Chronic stress has a degenerative effect on PFC structure and functioning (Arnsten, 2009, 2015; McEwen and Morrison, 2013) and seems to adversely affect its plasticity (McEwen et al., 2012). A study by Zhang et al. (2014), that builds on the correlational work of Nagai and others (Nagai et al., 2004, see Critchley and Garfinkel, 2015, for a review) shows a causal involvement of ventromedial PFC in physiological arousal: when ventromedial PFC activity goes up, electrodermal activity (skin conductance level) goes down. In other words: prefrontal structures suppress stress. A conclusive review by the international behavioral neuroscience meeting (Radley et al., 2015) stated the negative effects of stress on the plasticity of the limbic network (amygdala, hippocampus and PFC) and its pivotal role in the etiology of aforementioned (mental) health conditions.

In sum, (chronic) stress is a significant negative mediator in all of the domains that benefit from ContAct. It is here proposed that these beneficial effects occur by (chronic) stress relief as a result of vagal dominance by rVNS. In other words: breathing exercises produce stress relief (Lee et al., 2003; Pramanik et al., 2009; Perciavalle et al., 2017). We have also already indicated that chronic and high levels of stress are negatively related to executive functions and PFC functioning. Next, we will show that there is also a positive relation between vagal tone, CAN areas and the executive functioning network, as predicted by the neurovisceral integration model, and between changes in CAN and ContAct practice as predicted by the rVNS model of ContAct.

### CAN: Regulated Emotion and Enhanced Cognition

The link from VN to PFC (Ter Horst and Postema, 1997; Wager et al., 2009a,b) is a critical element of the CAN in mediating rVNS effects of ContAct onto executive functions. Likewise, projections into limbic parts of the CAN allow ContAct to enhance positive affect via rVNS. If these projections are actually used, vagal tone should have a positive correlation: (i) with executive functions or PFC activation and (ii) with emotional control or medial PFC activation. See Thayer and Lane (2000) for the CAN network as adapted in the original neurovisceral integration model.

#### HRV, Cognitive Control and PFC

There indeed is an association between HRV and executive functions, as first shown by Thayer and Fischer (2009), especially in emotional control: HRV predicts inhibition of attention to emotional stimuli (Park et al., 2012, 2013), it shows a positive relation to attentional control and negative relation to risk aversion in anxiety (Ramírez et al., 2015), predicts attentional lapses (Williams et al., 2016), and it is involved in cognitive inhibition, proactive cognitive control (Capuana et al., 2012, 2014) and emotional inhibition of conditioned fear (Wendt et al., 2015). A recent meta-analysis also supports a relationship between HRV and cognitive control: executive functioning, pooled across the subdivisions of Miyake et al. (2000), showed a positive average association with HRV level, though the authors note a strong publication bias (Zahn et al., 2016). The effects can especially be observed in cognitively demanding settings. A brain imaging study shows that functional connectivity of the amygdala and medial PFC are associated with higher HRV in both younger and older people (Sakaki et al., 2016).

The link between HRV and the PFC seems to be very direct: they share a common genetic background (Thayer et al., 2009) and HRV and executive functions show a similar ontogenetic developmental trajectory; going up until early adulthood and going down again with advancing age (Umetani et al., 1998; Zelazo et al., 2004). This is expected if VN and PFC form a single system: CAN. Another clue to the involvement of VN in executive functions comes from the work of van der Molen (2000) into the development of inhibitory control. During successful cognitive inhibition of action representations, heart rate deceleration can be observed, after which heart rate goes up again (Schel et al., 2013). As we have seen, heart rate slowing is under direct control of VN, indicating vagal dominance during employment of cognitive control.

#### Changes in CAN Regions Through ContAct

As we hypothesize that in ContAct rVNS is responsible for the emotional and cognitive enhancement by changes in CAN, studies looking at functional and structural changes in the brain in practitioners should show these changes along this whole network—in the limbic system and executive functioning network; in the levels of the updated neurovisceral integration model (see Smith et al., 2017). Studies on ContAct practice have shown this for the limbic part of CAN. For example, a decrease in volume and activity has been observed in the amygdala (Hölzel et al., 2010; Tang et al., 2015) and in the hippocampus (Luders et al., 2009, 2012c) in practitioners of different styles of meditation (both FA and OM). Insular cortex and posterior cingulate also increase in activity and volume in the same populations (Lazar et al., 2005; Hölzel et al., 2011a,b; Kirk et al., 2011; Luders et al., 2012b; Tang et al., 2015). The practice of yoga shows the same pattern (Froeliger et al., 2012; Desai et al., 2015), while a TCC study shows the most significant morphological changes in the insula and dorsolateral PFC (Wei et al., 2013).

The ACC is a limbic structure, but is also part of executive functioning network and active in cognitive control, and is central in CAN. Notably its dorsal part has been implicated in autonomic control, as it modulates cardiovascular stress responses (Critchley et al., 2003). Following the previous argument, ACC should also be implicated in imaging studies of ContAct efficacy, and indeed, functional and structural enhancement in ACC has been reported in meditation styles and in mind-body exercises (Cahn and Polich, 2006; Tang et al., 2009, 2010, 2015; Hölzel et al., 2011b; Xue et al., 2011; Wei et al., 2013). The frontal end stations of CAN also show predicted structural changes: PFC gray matter density is increased by diverse meditation styles and mind-body exercises (Lazar et al., 2005; Luders et al., 2009, 2012a; Lutz et al., 2015; Hölzel et al., 2011a; Froeliger et al., 2012; Tang et al., 2015; Wei et al., 2013; Yin et al., 2014; Desai et al., 2015). In sum, there is a large overlap between the brain regions changed by ContAct—amygdala, hippocampus, insula, ACC and multiple areas of the PFC—and those identified in CAN. Note, however, that these areas have been implicated in behavioral studies with very diverse tasks and contexts—not only in ContAct.

#### Default Mode and Executive Network Plasticity by VNS

In general, brain connectivity seems to increase by ContAct practice across multiple projections, commissures and associatedsociative networks, as shown by several diffusion tensor imaging studies (Luders et al., 2011, 2012b). Meditation practice (and trait mindfulness) is associated with greater connectivity between the executive, DMN and salience networks specifically (Brewer et al., 2011; Hasenkamp and Barsalou, 2012; Doll et al., 2015). Notably the DMN is implicated in neuroimaging studies among ContAct practitioners. DMN is active when external stimulation and work demand is low. Its main hubs are medial PFC, posterior cingulate cortex and parahippocampal region (Raichle et al., 2001); the last of which is believed to operate as the hub between DMN and limbic areas (Ward et al., 2014). The role of DMN in cognitive control can be seen as opposing that of the executive network; lapses of attentional control (i.e., mind wandering) are contingent on DMN activity over executive network activity (Gratton et al., 2018), in this way DMN can be viewed as a “task-negative” network (Fox et al., 2005). Studies on changes in connectivity by ContAct experience show deactivation of DMN hubs (i.e., posterior cingulate and medial PFC) and decreased functional connectivity between these hubs. At the same time the functional connectivity between the DMN and executive networks goes up (Brewer et al., 2011; Hasenkamp and Barsalou, 2012). This mirrors what is consistently found in imaging studies that apply VNS to individuals with (treatment-resistant) depression.

Depression is associated with a disrupted DMN, particularly: hyperactivity and hyper-connectivity among DMN hubs, as well as hyper-connectivity between DMN and limbic system, and hypo-connectivity between DMN and executive network (Drevets et al., 2008; Gong and He, 2015; Kaiser et al., 2015). Clinical trials employing chronic VNS in patients with depression show a normalization of this etiology, obtaining results very similar to ContAct practice. One study on patients with depression not responding to regular treatment showed increased metabolism in the DMN hub ventromedial PFC (Pardo et al., 2008). While a similar study reported decreased activity (regional cerebral blood flow) in another DMN hub (posterior cingulate) and in the limbic system (insula), concurrently increasing activity in dorsolateral PFC of the executive network (Kosel et al., 2011). Another imaging study (on epilepsy), reports a decrease in regional cerebral blood flow in the DMN hub parahippocampus, as well as in the hippocampus and the thalamus by chronic VNS (Van Laere et al., 2002).

The few tVNS studies so far show a similar pattern as those obtained with VNS. One study of major depression reports that tVNS decreases the resting-state functional connectivity between main DMN hubs and parahippocampus—the DMN hub that connects to the limbic system—and anterior insula (Fang et al., 2016). Contrarily, it increases the resting-state functional connectivity of DMN with the precuneus and orbitofrontal cortex (executive network). In addition, all these connectomic changes were associated with reductions in depression severity. A fMRI study in a normal population shows that tVNS can acutely reduce activity in DMN hubs: parahippocampal and posterior cingulate (Kraus et al., 2013). There are also indications that tVNS produces changes in the executive network itself. Badran et al. (2018) are the first to show increases in metabolic activity between the main hub axis of dorsolateral PFC and ACC by tVNS. Another tVNS study produces changes between the executive network and limbic system, by decreasing functional connectivity between rostral ACC and medial hypothalamus in depression, all associated with clinical improvement (Tu et al., 2018). Somewhat paradoxically, a study on patients diagnosed with major depressive disorder showed a decrease in symptoms due to tVNS, but combined with an increase in resting-state functional connectivity between the amygdala and dorsolateral PFC, so between the limbic and executive systems (Liu et al., 2016).

Concluding: it has clearly been shown that activity in afferent branches of VN can affect areas and networks in the CNS, both acutely (e.g., by tVNS) and chronically (e.g., by chronic VNS). This notably affects the DMN, which is a critical CAN level in the latest version of the neurovisceral integration model (see Smith et al., 2017 for details). Central changes as a result of ContAct practice within DMN and between DMN and executive network are practically identical to those observed by (t)VNS studies. This makes vagal involvement and thus the mechanism of rVNS highly likely in producing these neurobiological effects and the concomitant improvements in cognition and affect. Concretely stated: DMN deactivation and increased DMN-executive network connectivity is caused by rVNS and will lead to improvements in cognitive control (e.g., cognitive inhibition) and performance monitoring.

## Discussion

We have shown that ContAct practices, though diverse, have a number of components in common that can explain their efficacy in individual physical health, mental health and cognition. Furthermore, one of these components: breathing techniques, is a prime candidate to explain the complete pattern of results, notably in the stress-related domain. We have further provided a neurophysiological model in which slow respiration and extended exhalation stimulate VN via two possible routes: rVNS. This results in PNS over SNS dominance, structural and functional changes in higher cortical areas through autonomic projections, and is thus responsible for aforementioned effects.

In these claims, one of the main arguments for the rVNS model of ContAct concerns the dovetailing between specific functions of VN with the pattern of effectivity shown by diverse ContActs; providing beneficial effects on cardiopulmonary fitness, immune function, psychological health, stress, anxiety and executive functions. The neurophysiological link between the two can be found in vagal tone: the existent relationship between the aforementioned functions and conditions with HRV and that of HRV with the VN. Evidently HRV is then an index of adaptability in a broad sense.

We realize that there might be more common factors involved in ContAct interventions than we have covered and categorized here. There also might be unique components to particular traditions, as well as emergent properties of specific combinations of components; almost all ContAct interventions are multi-modal. For example, many of the covered traditions are not practiced in isolation, but in group sessions. Social, and even physical, contact could be a factor in relieving stress and in alleviating depression. Though we do not discount the other factors covered and those possibly left out, we believe that respiration style and vagal functioning fit the evidence best, and following Occam’s razor, it stands out as the most parsimonious of explanations. However, also rVNS might benefit from a specific combination. For instance, combining rVNS with affect training: exposure and reappraisal procedures could be strengthened by the concurrent body relaxation brought on by rVNS, the biofeedback would weaken the stress response and negative emotion brought up by an aversive or traumatic memory.

Following the observation on the multi-modality of ContAct, we maintain that many null-findings and conflicting results in the literature could be ascribed to the presence or absence of particular effective components. For example, a yoga class only focusing on stretching and shifting positions might not have any executive functions benefits other than those stemming from some form of relaxation, but does show changes associated with mild exercise. Studies performing systematic analyses that compare functional ContAct elements, based on concrete predictions, are therefore sorely needed. Reported findings that show controlled breathing increasing SNS activity further underline the importance of making clear what kind of techniques are employed. This includes reporting on the exact instructions given and controlling for compliance to these instructions. Our predictions are only valid as far as interventions result in slow, deep (diaphragmatic) breathing—not in other breathing styles, such as fast and deep breathing during physical exercise. Thus, the described beneficial effects on health and cognition are predicted to occur more in ContActs with breathing exercises stressing relatively short inhale (SNS controlled) and long exhale (PNS controlled), than in ContActs that do not emphasize this distinction.

Some ContAct practitioners might proclaim that their particular tradition (notably FA) does not involve any breathing exercises. That the exercises only instruct to pay attention to the breath, and not to modulate respiration in any way; that instructions to change anything in breathing patterns are absent. They might also state that the breath is only one of many foci. For example: it could be a visual focus, such as a flickering candle flame or verbal, as in a mantra. But the fact is, that across these diverse FA traditions it usually is not another kind of focus, it usually is the breath, and we maintain that this is not arbitrary. As previously stated, we maintain that it is unlikely that focusing on the breath does not affect respiratory patterns. In our view, directed conscious awareness to breathing will slow respiration in expert and layman alike, through direct and indirect experience with different breathing exercises and the implicit or explicit idea, an ideomotor representation if you will, what it should be like to meditate: meditating involves relaxed breathing. Furthermore, the rhythm of auditory instructions is in a slower pace than normal breathing, thereby slowing respiration as well. Of course, these are assumptions that should be tested in further experiments. But, if attention does slow respiration, this makes all these traditions fall under the explanatory umbrella of the rVNS model of ContAct.

So far, the picture painted from rVNS has been optimistic. However, there might be circumstances and doses where no beneficial effects can be expected. For example: in chronically stressed individuals vagal activation might have such a high threshold that rVNS will have no noticeable effect; they might prove resistant to the intervention. rVNS might even have adverse effects, such as overstimulation. In a condition known as vasovagal syncope, vagal efferents reduce heart rate to such a degree that blood pressure drops to dangerous levels, resulting in fainting and symptoms of chronic fatigue. As indicated by VNS studies showing bronchoconstriction, stimulation might be dangerous for pulmonary pathology, such as chronic asthma (Hajnšek et al., 2010). However, studies varying VNS voltage suggest that strength of stimulation could make the difference between beneficial or detrimental (Hoffmann et al., 2012). Respiratory VNS might be as beneficial and healthy as the individual baselines (e.g., autonomic balance) allow. By extension, these dangers could be present for ContAct practices as well. But as far as we know, there are no studies on adverse effects produced by ContActs. This does not deny their existence as the absence could be a result of publication bias.

On a similar note: higher vagal tone HRV is not always better. Much like arousal levels optimal levels of HRV for physical and mental functioning might follow an inverted U-shape, as in the Yerkes-Dodson law (Yerkes and Dodson, 1908). Individuals with a resting HRV at the right side of their personal curve might actually present adverse effects if HRV levels are further increased, autonomic balance shifting too far away from the sympathetic. In contrast, individuals with HRV levels falling at the left end of that curve might benefit the most from increasing HRV (by rVNS), being “parasympathetically compromised.” Also, there might be differences between populations in the shape of this curve and there might be different curves for different VN functions. For example, as first reported by Wang et al. (2005), but see Hill et al. (2015) for a review, African Americans’ resting HRV is on average higher than that of Caucasian Americans, while the prevalence of cardiovascular disease is higher in African than in Caucasian Americans. This is counterintuitive if HRV is seen as a pure measure of vagal tone. At the same time, the relationship with anxiety and depression does follow the predicted direction: African Americans suffer less from these conditions than Caucasian Americans (Breslau et al., 2006). Further research has to address the question whether there is an inverse relationship between HRV and cardiovascular disease in this population, whether relatively higher HRV for this population is not optimal HRV regarding cardiopulmonary function, giving room for further enhancement, or perhaps whether this is a HRV methodological artifact.

In light of the necessity to report the specific instructions and exercises in every intervention study, mention has to be made of mindfulness meditation. When we and many other authors report on mindfulness meditation, this usually refers to two clinical programs: mindfulness-based stress reduction and mindfulness-based cognitive therapy. Both of these are multi-modal interventions, in which not only mindfulness meditation and other meditation techniques play a role, but also physical exercises (some form of yoga), mental strategies, and in mindfulness-based cognitive therapy: cognitive-behavioral therapy. Most studies that report on the effects of mindfulness or mindfulness meditation, that use these programs as interventions, should therefore strictly speaking not be taken as “pure” mindfulness meditation; subsequently, caution is advised in interpreting result.

We want to note that HRV (respiratory sinus arrhythmia) as a valid measure of vagal tone, also maintained by Thayer and colleagues, is not without its critics (Grossman and Taylor, 2007). Grossman and colleagues have shown in their experiments that under different respiration conditions, respiratory sinus arrhythmia does not reflect changes in vagal tone accurately (Kollai and Mizsei, 1990; Grossman and Kollai, 1993; Taylor et al., 2001). They maintain that HRV measures should always be controlled for by respiration. Since these studies, there has been limited follow-up of these criticisms, as can be seen in a recent review of HRV methods (Laborde et al., 2017). We believe this issue should be addressed resolutely. More neuroscientific experiments directly assessing the relation between the different HRV measures and vagal tone are needed. As far as the implications for the rVNS model of ContAct: it includes assumptions made by the polyvagal and the neurovisceral integration accounts; and it loads evidence for the existence of rVNS on those assumptions. However, we maintain that HRV does not need to be a “pure” measure of vagal tone for it to be useful as a measurement—a relational representation is enough. But even if respiration confounds on vagal tone are insurmountable, making HRV unusable as its measure, this will not affect the core assumptions of our model and the predictions it makes. In the future, other measures of SNS/PNS activity and balance should be developed. In any case, the rVNS model of ContAct still provides testable and falsifiable predictions.

Though we are not able to definitively prove a causal link between breathing, VNS and improvements in body and mind, we believe we have provided ample evidence suggesting the existence of this link, largely by providing overlapping patterns of specific phenomena. Empirical studies need to put our hypotheses to the test. Furthermore, other concrete neurobiological mechanisms for the systems described in this work need to be proposed and charted by experimental studies, as prescribed for the field by Thayer et al. (2011). Studies on different respiration styles, using psychophysiological measures for PNS and SNS activity and tasks assessing executive functions acutely and longitudinally could provide concrete tests of our hypotheses. Imaging studies mapping structural and functional changes in the CAN following rVNS are critical for our hypotheses on cognitive functioning, especially: changes in DMN and executive network mirroring VNS results are expected. Other indirect tests include experiments comparing ContAct interventions with (t)VNS manipulations. As an established phenomenon, studies of rVNS dose-response relationships, with personal baseline levels, could follow. What is the “vagus code” of rVNS (Kwan et al., 2016)? What are observed differences caused by specific modes of stimulation? For instance, electrical VNS and behavioral rVNS differ also in laterality: unilateral vs. bilateral stimulation, which might produce different effects (and strength of stimulation).

Lastly, as for the reasons why breathing techniques have gone pretty much unnoticed as a mechanism of ContAct efficacy, while being so prevalent and well-known, we propose that it perhaps has to do with this prevalence: it is such an unremarkable fact, so plainly observable and a starting point of practice. This could be coupled with a tendency to focus on “higher” levels of consciousness among practitioners, and on higher level processes and structures in cognitive neuroscientific research. Relaxation might also be assumed and uninteresting, breathing exercises are automatically factored in, something for novices, hardly remembered by the expert. We hope that through this work future research on ContActs will recognize and study breathing techniques as an effective component, and that neuroscience will focus on rVNS respiratory patterns as potential cognitive and (mental) health enhancers.

## Author Contributions

RG conceived the presented idea, developed the model, posited possible mechanisms and wrote the main drafts. GB supervised the project, and edited and rewrote manuscript drafts. All authors contributed to the final version of themanuscript.

## Conflict of Interest Statement

The authors declare that the research was conducted in the absence of any commercial or financial relationships that could be construed as a potential conflict of interest.

## Abbreviations

CAN: Central Autonomic Network
ContAct: Contemplative Activity, such as meditation, mindfulness and yoga.
FA: Focused Attention meditation
OM: Open Monitoring meditation
PNS: Parasympathetic Nervous System
rVNS: respiratory Vagal Nerve Stimulation
SNS: Sympathetic Nervous System
TCC: Tai Chi Chuan
VN: Vagus Nerve.
